# Land carbon models underestimate the severity and duration of drought’s impact on plant productivity

**DOI:** 10.1038/s41598-019-39373-1

**Published:** 2019-02-26

**Authors:** Hannah R. Kolus, Deborah N. Huntzinger, Christopher R. Schwalm, Joshua B. Fisher, Nicholas McKay, Yuanyuan Fang, Anna M. Michalak, Kevin Schaefer, Yaxing Wei, Benjamin Poulter, Jiafu Mao, Nicholas C. Parazoo, Xiaoying Shi

**Affiliations:** 1 School of Earth and Sustainability, Northern Arizona University, P.O. Box 4099, Flagstaff, AZ 86011-5694 USA; 2 Woods Hole Research Center, Falmouth, MA 02540 USA; 3 Jet Propulsion Laboratory, California Institute of Technology, 4800 Oak Grove Dr., Pasadena, CA 91109 USA; 4 Department of Global Ecology, Carnegie Institution for Science, Stanford, CA 94305 USA; 5 National Snow and Ice Data Center, Cooperative Institute for Research in Environmental Sciences, University of Colorado, Boulder, Colorado USA; 6 Environmental Sciences Division, Oak Ridge National Laboratory, Oak Ridge, TN 37831 USA; 7 NASA Goddard Space Flight Center, Biospheric Sciences Laboratory, Greenbelt, MD 20771 USA; 8 Environmental Sciences Division and Climate Change Science Institute, Oak Ridge National Laboratory, Oak Ridge, Tennessee 37831-6301 USA

## Abstract

The ability to accurately predict ecosystem drought response and recovery is necessary to produce reliable forecasts of land carbon uptake and future climate. Using a suite of models from the Multi-scale Synthesis and Terrestrial Model Intercomparison Project (MsTMIP), we assessed modeled net primary productivity (NPP) response to, and recovery from, drought events against a benchmark derived from tree ring observations between 1948 and 2008 across forested regions of the US and Europe. We find short lag times (0–6 months) between climate anomalies and modeled NPP response. Although models accurately simulate the direction of drought legacy effects (i.e. NPP decreases), projected effects are approximately four times shorter and four times weaker than observations suggest. This discrepancy between observed and simulated vegetation recovery from drought reveals a potential critical model deficiency. Since productivity is a crucial component of the land carbon balance, models that underestimate drought recovery time could overestimate predictions of future land carbon sink strength and, consequently, underestimate forecasts of atmospheric CO_2_.

## Introduction

Terrestrial ecosystems modulate the increase in atmospheric CO_2_ from anthropogenic emissions by sequestering carbon in vegetation and soils. Although the strength of the residual land carbon sink has increased over recent decades^1^ due to a combination of effects such as CO_2_ fertilization^2^, high latitude warming^3^, and an increase in growing season length^4^, the future strength of land carbon uptake is highly uncertain, with some projections indicating that the global terrestrial biosphere will switch from being a net sink to a net source of carbon to the atmosphere by 2100^5–8^. One major source of uncertainty stems from the unknown impact of more frequent and severe droughts^9,10^ on terrestrial carbon dynamics^11,12^. Droughts drive changes in land sink interannual variability^13–15^, generally reducing net carbon uptake in the affected region by decreasing plant productivity and increasing mortality^16,17^. Severe drought can temporarily shift ecosystems from a net sink to a net source of carbon on the order of months^16,18^, and some studies predict that future climate regimes will cause certain regions to transition over decades from being a carbon sink to a permanent carbon source^19,20^.

**Figure 1:**
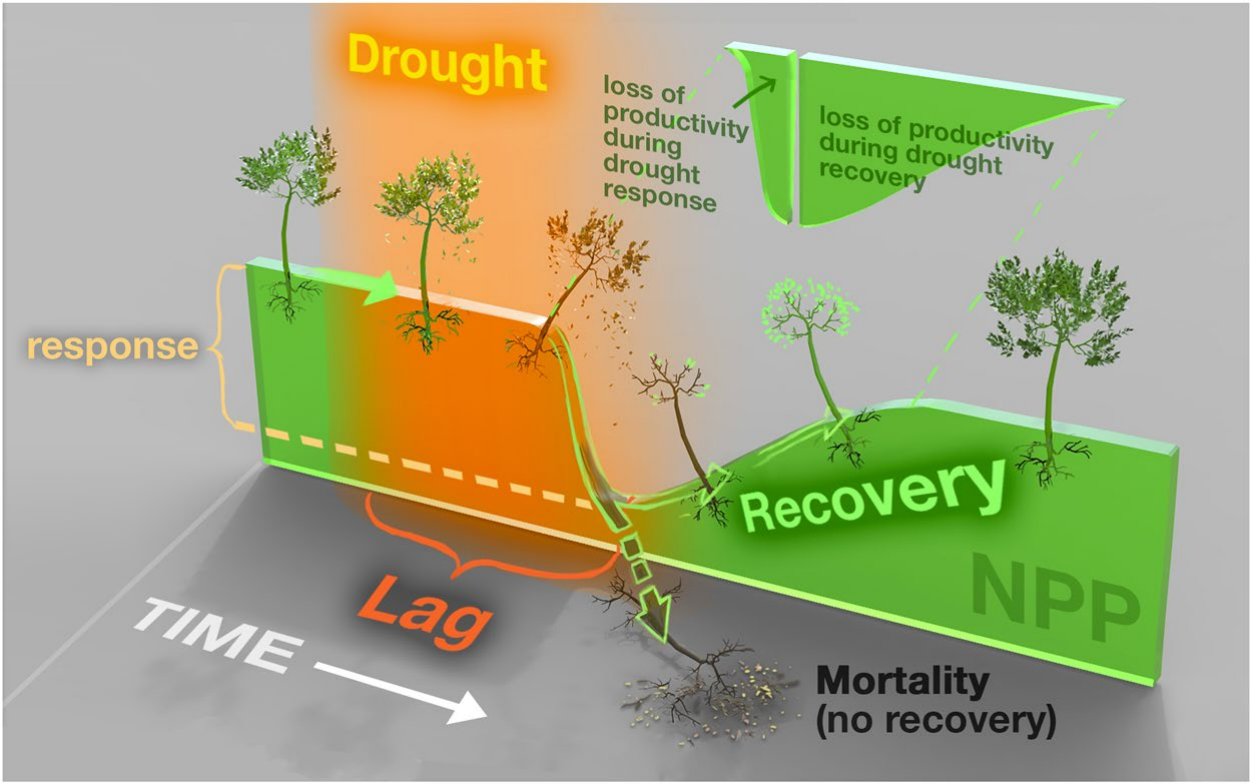
Schematic depicting how severe drought can impact NPP. Starting on the left, with time progressing to the right, this example ecosystem begins at typical vegetation growth rates (the height of the green surface). Drought (the orange shaded period) can decrease productivity even after the return to nominal climate conditions. Drought response is the initial depression in growth following the onset of a drought, indicated here as the depth of the trough in NPP. Lag time is the time between the onset of drought and the drought response. Recovery describes the amount of time required to recover normal growth rates. Drought legacy effects correspond to the magnitude of NPP depression during drought recovery. The cut-out wedge illustrates the total loss of NPP, relative to typical productivity - during the drought event itself and through the end of drought recovery. Alternatively, vegetation may be unable to recover from drought and, instead, follow the path to mortality. Note that the relative magnitudes of these variables (e.g. larger cumulative loss of productivity during drought recovery than the drought response) depend on factors such as the drought’s characteristics and the type of vegetation; this illustration provides an example of drought’s impact on productivity rather than the rule. Illustration by Victor O. Leshyk. Printed with permission by Victor O. Leshyk under a CC BY open access license (https://creativecommons.org/licenses/by/4.0/).

In addition to reductions in carbon uptake concurrent with drought (i.e., drought response, see Fig. 1), ecosystems often experience lower tree growth rates and higher tree mortality rates for several years following the return to nominal climate conditions^21–23^. These delayed ecosystem responses, or drought legacy effects, may significantly influence the interannual variability of carbon cycling and the magnitude of long-term terrestrial carbon storage. For instance, if ecosystem recovery time (see Fig. 1) exceeds the drought return interval, ecosystems may become more vulnerable to future drought, since recurrent drought can cause a progressive loss of vegetation drought resilience^24–26^. Ecosystem degradation and the subsequent weakening of land carbon sinks from recurrent drought will likely become more prevalent in the future as the frequency of extreme droughts increases.

Although drought legacy effects could play a crucial role in carbon cycling, it is unclear whether current models accurately simulate the vegetation’s response to and recovery from drought. There is evidence that models from the Coupled Model Intercomparison Project Phase 5 (CMIP5)^27^ overestimate the effect of dryness on gross uptake while underestimating how long productivity is sensitive to climate anomalies^28^ and underestimate the magnitude and timescales of vegetation drought recovery^21^. However, the coupled nature of these models can make it difficult to isolate and compare the impact of particular extreme events across models. This is because there is substantial variability in climate conditions such as temperature, precipitation regimes, and global radiative forcing across the CMIP5 ensemble^29–31^. Additionally, characteristics of extreme climate indices and events (e.g. maximum monthly temperature, warm spell duration, consecutive wet days) vary widely across models^32,33^.

In order to isolate the simulated response of the land surface to climate extremes, we evaluate offline process-based terrestrial biosphere models (TBMs) driven with prescribed climate and a standardized simulation protocol. We identify drought using the drought metric Climatic Water Deficit (CWD), defined as precipitation minus potential evapotranspiration, and assess vegetation productivity response by examining changes in modeled NPP. Evaluating the relationship between CWD and NPP rather than gross productivity (as in^28^) provides a better, albeit incomplete, picture of drought’s impact on carbon sinks. We quantify both the magnitude and timescales of several phases of modeled drought response and recovery in terms of productivity (lag, response, and recovery; Fig. 1) that observational studies suggest should be present^21,26,34–36^. We further assess how these features of drought response and recovery vary across land cover types and regional climate conditions. Finally, we compare modeled drought recovery with observed drought recovery to determine whether models simulate realistic long-term drought impact on vegetation growth.

## Results and Discussion

**Figure 2:**
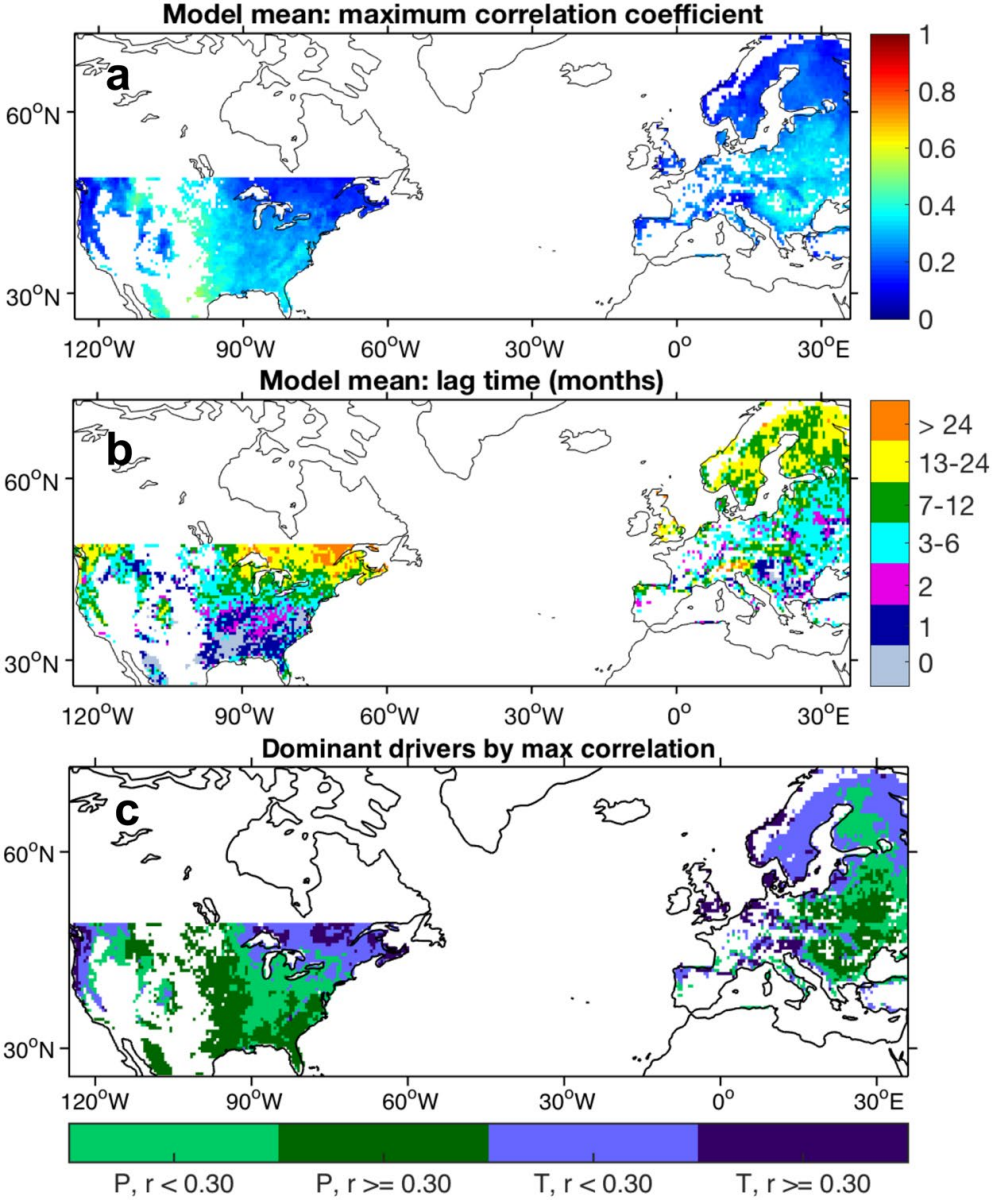
NPP sensitivity to CWD anomalies. (**a**) Mean across models of maximum correlations between monthly de-seasonalized (grid cell monthly means removed) CWD and monthly de-seasonalized NPP (maximum correlation out of 61 correlations calculated per grid cell, with an offset of 0–60 months between NPP and CWD). (**b**) Lag time (offset) corresponding to the maximum correlation. Values have been binned. Averages for each grid cell were calculated using only significant correlations (FDR-adjusted *P* < 0.05) and their corresponding lag times per model. (**c**) Dominant climate driver (P = precipitation, T = temperature) of NPP, determined by maximum correlation, *r*.

The first feature of drought response and recovery is the lag time, the timescale associated with the initial reduction in NPP following the onset of drought (Fig. 1). We quantified this using the optimized time-lagged correlations between monthly CWD anomalies and NPP anomalies (see *Methods*). Sub-annual lag times between CWD anomalies and NPP response indicate a strong immediate modeled NPP response to drought (Fig. 2b). Although lag times varied substantially across the spatial domain, the majority of the domain experienced short lags (0–6 months). The longest response times corresponded to the lowest sensitivities and were prevalent at higher latitudes. In these regions, temperature and radiation are the limits on productivity^37^ (Figs S1 and S2). Because CWD is more dependent on precipitation than temperature, high lags in higher latitudes are likely due to the weak relationship between CWD and NPP. The highest correlations (*r* ~ 0.5) occur in the central US. The western US, a region limited by both water and temperature, surprisingly exhibits weak NPP sensitivity to moisture anomalies across all climate metrics. Since this is a region that has experienced frequent drought, one potential cause of this weaker sensitivity is the decoupling of growth and climate following drought (i.e. reduced growth plus rebounding moisture), characteristic of drought legacy effects. It is unclear, however, how much drought legacy influences low correlations between CWD and NPP relative to other factors, such as radiation or growing season length.

**Figure 3:**
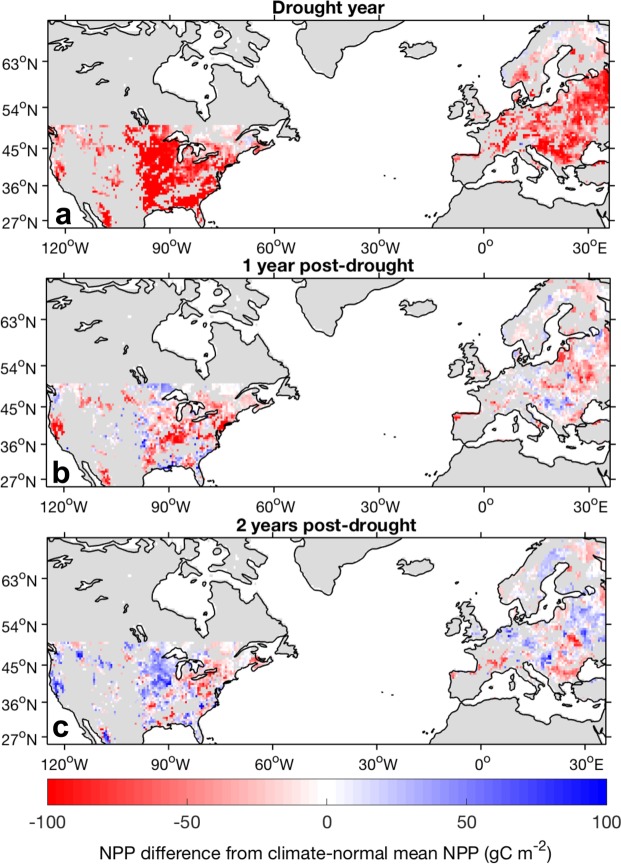
Spatial maps of the model mean NPP anomalies. Anomalies are defined as the difference from the climate-normal NPP of each grid cell (the average NPP across years in which −1 ≤ CWD ≤ 1). NPP anomalies correspond to (**a**) the drought year, (**b**) one year post-drought, and (**c**) two years post-drought. Grey areas indicate pixels that experienced no drought events or that were excluded from analysis (outside spatial bounds, unforested grid cells).

Differences between annual NPP and climate-normal mean NPP, the average NPP across normal hydrological conditions (i.e. −1 ≤ CWD ≤ 1), revealed a strong drought response, along with rapid recovery and relatively weak drought legacy effects on growth. Here, we quantify the drought response as the initial drought-induced reduction in NPP relative to the climate-normal mean NPP and drought legacy effects as subsequent reductions in NPP until productivity returns to climate-normal values (Fig. 1). Across all forested pixels, drought immediately decreased NPP in the drought-year by an average of 75.7 gC m^−2^ relative to the climate-normal mean NPP. Integrated over the region of analysis, this amounts to a total NPP reduction of 0.55 PgC, or ~14% of the climate-normal mean NPP. In the first post-drought year, NPP partially rebounded with an average NPP reduction from the climate-normal mean of 18.0 gC m^−2^, or 0.13 PgC across the region. There is considerable spatial heterogeneity across these annual NPP anomalies. In the drought year and first post-drought year (Fig. 3a,b), negative NPP anomalies were prevalent throughout the US and Europe, with larger negative NPP anomalies in more productive regions. In most regions, however, NPP fully recovered in the second post-drought year.

**Figure 4:**
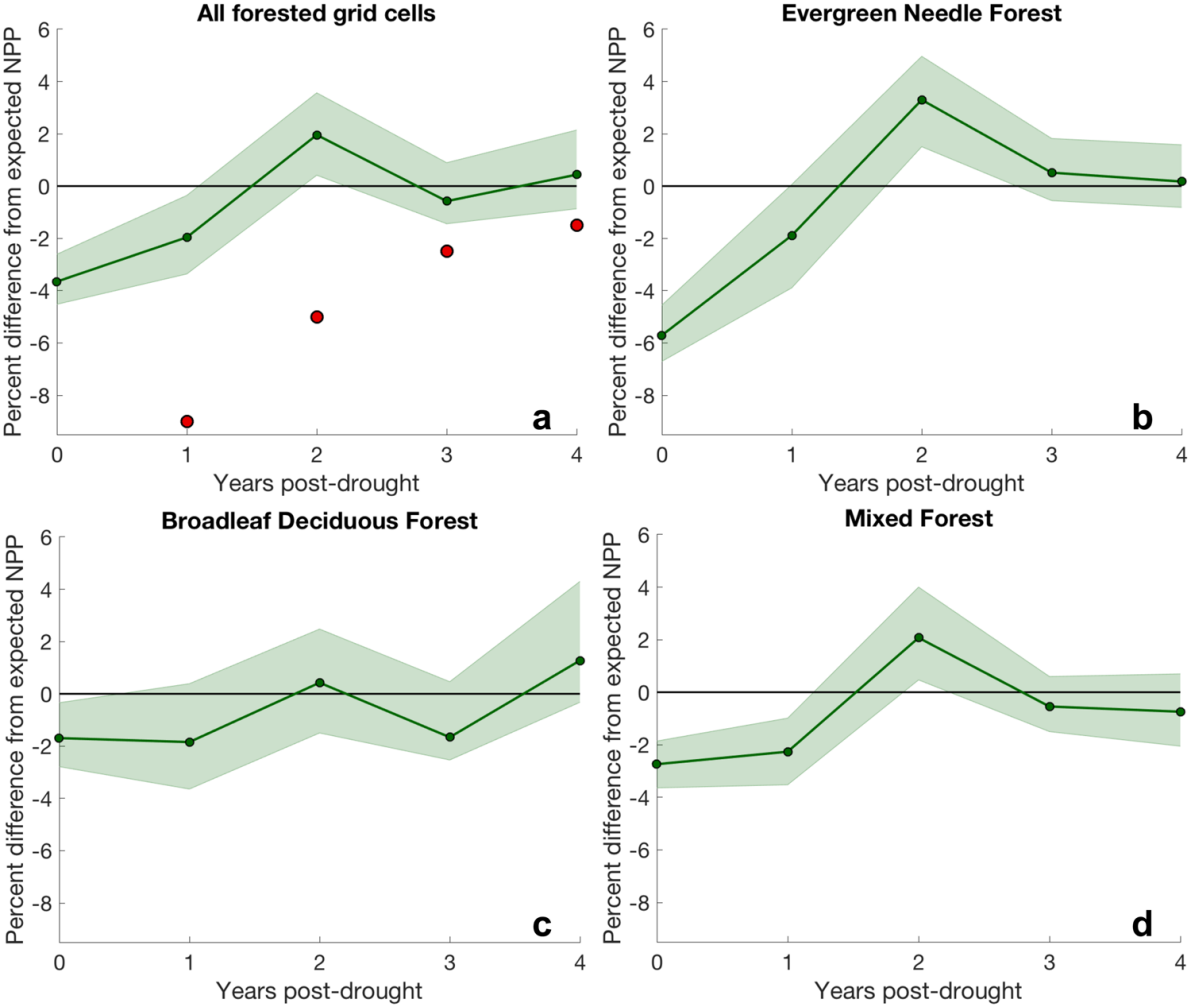
Percent difference between NPP and expected NPP based on CWD. Differences are displayed for the drought year and four years following drought across (**a**) all forested grid cells with the region of analysis, (**b**) evergreen needle forest, (**c**) broadleaf deciduous forest, and (**d**) mixed forest. In (**a**), the red circles indicate an observational benchmark derived from tree rings^21^. Green shaded regions indicate the 95% confidence intervals from bootstrapping (n = 5000) the individual model means (a measure of model spread).

We found no clear relationship between mean climate (represented by mean annual precipitation) and the magnitude of drought response or timescale of recovery (Fig. S3), despite evidence suggesting that productivity in semi-arid regions exhibits greater drought impact and longer recovery times than productivity in more humid regions^21,38,39^. Although not included in this study, soil properties can also regulate climate’s impact on vegetation water use efficiency^40^, and this may play a role in the lack of relationship between mean climate and drought recovery. For models that dynamically calculate LAI, drought-induced changes in LAI may explain some of the modeled drought legacy effects evident through NPP. Relative to the climate-normal mean LAI, models on average exhibited the largest decreases in LAI in the first post-drought year (up to 20% reductions in LAI, Fig. S4), consistent with a reduced capacity for leaf-level photosynthesis across the spatial domain and potentially reduced NPP. However, spatial patterns associated with large decreases in NPP do not necessarily align with large decreases in LAI, and there are no significant differences in productivity recovery from drought between deciduous and evergreen forests (see Fig. 4). Furthermore, the models that best capture extended drought recovery (Fig. S5) do not simulate large LAI reductions in the first year following drought (individual model LAI results not shown). Thus, it is not immediately clear what factors are responsible for the spatial distribution of NPP drought legacy effects.

To evaluate how consistent modeled drought legacy effects are with observations, we follow the methods of Anderegg *et al*.^21^ and quantify drought-induced NPP anomalies as the difference between NPP and the NPP anticipated from climate conditions based on a linear relationship between CWD and NPP (see *Methods*). We found that models simulate rapid NPP recovery from drought, with weaker drought legacy effects on growth than the observations suggest (Fig. 4). Across all forested grid cells (Fig. 4a), in the first post-drought year, NPP was depressed an average of 2% relative to the NPP expected based on CWD. This is about one quarter of the growth decline suggested by observations (9% reduction in NPP). Although models exhibited considerable variability in first year post-drought legacy effects, ranging from a 5.9% reduction to a 3.5% increase in NPP relative to CWD-predicted values (Fig. S5), even the most extreme NPP depression fell short of the corresponding observed growth reduction. In the second year post-drought, modeled growth rebounded strongly, whereas observations suggest that growth should remain depressed. To explore the variability of modeled drought recovery and to examine potential features obscured when using the mean, we identified different percentiles of drought legacy effect across the ensemble of models. These results indicate that modeled legacy effects exhibit a substantial amount of variability, ranging from nearly 30% reduction in NPP to a nearly 30% increase relative to NPP expected based on CWD (Fig. S6). The 25^th^ percentile roughly corresponds to the magnitude of observed drought legacy effects in the first and second years following drought. Thus, while models on average appear to underestimate both the size and persistence of drought legacy effects, their range of variability includes realistic legacy effects.

While models underestimate the legacy impact of drought events on NPP, they may be overly responsive to the drought itself. Across all forested regions and each forest type, the drought year yields either the greatest deviations from expected NPP based on CWD (Fig. 4a,b,d) or deviations comparable with the first post-drought year (Fig. 4c). These drought year NPP reductions are more consistent across MsTMIP models (most narrow 95% confidence intervals) than any post-drought NPP deviations. Since expected NPP derived from CWD already accounts for the depression in growth due to water limitation, considerable departures from expected NPP in the drought year suggest that models may be overly sensitive to the drought event itself. This is consistent with the findings of other studies examining the drought response of CMIP5 models^28^ where anomalies of modeled leaf area index (LAI) were more highly correlated with a drought metric than are observed LAI, suggesting that modeled productivity may be unrealistically responsive to climate anomalies. Nevertheless, because the observational benchmark does not report growth reduction in the drought year and instead focuses on post-drought years, we cannot formally assess model performance in the drought year.

The dominant forest type in a given region impacts the immediate drought response simulated by models, but does not have a strong influence on the magnitude or duration of projected drought legacy effects (Fig. 4). We define dominant forest type as the forest type with the highest percent cover within a grid cell (see Fig. S7 for map). Observations suggest evergreen needle trees should exhibit stronger, more persistent post-drought growth depression than broadleaf deciduous trees^21^. Although we do find that the drought response in evergreen needle forests was about 3 times stronger (Fig. 4b, ~6% reduction in NPP relative to expected NPP) compared to broadleaf deciduous and mixed forests (Fig. 4c,d, ~2% reduction in NPP relative to expected NPP), the legacy effects across all three types of forest all exhibited similar features - persisting for one year after drought and depressing NPP by about 2% relative to expected NPP. The similarities in legacy effects across forest types cannot be explained by low grid cell purity (i.e. a grid cell composed of several competing land types): restricting analysis to pixels containing high percentages of the given forest type yielded comparable results (Fig. S8). The similarity in simulated drought recovery across three distinct types of forests likely points to potential errors in the representation and/or parameterization of the physiological mechanisms responsible for diverse drought recovery characteristics across different types of vegetation.

The muted drought legacy effects simulated by models may be reasonable given their coarse resolution. The observational benchmark used is derived from individual tree ring chronologies, which preserve tree-level drought legacy effects directly from the source. However, tree ring chronologies may not adequately represent regional variability in carbon uptake due to drought. For instance, there is evidence that drought legacy effects vary drastically across species located within the same region (e.g. US Southwest^34^). While the benchmark is derived from a wide range of tree species, it still represents only a subset of variability in vegetation across the spatial domain. The extent to which plant-level variability translates into a drought recovery signal at regional scale (e.g., 0.5° grid) remains unclear. On the other hand, the benchmark could represent a conservative estimate of drought legacy effects on productivity, since it is based exclusively on trees that survived drought and, thus, ignores drought-induced tree mortality. Finally, precipitation-based CWD provides a more limited measure of plant water availability than soil moisture. Especially for vegetation rooted in deep soils^41^ or regolith^42^, CWD likely does not adequately represent climatic stress and may result in misleadingly weak drought legacy effects.

In summary, our results indicate that process-based models simulate strong drought-year growth reduction and underestimate the magnitude and duration of drought legacy effects on growth. The potential implications of this model behavior depend on whether the magnitude of modeled drought-year NPP reduction is realistic. Models may simulate the correct drought response while lacking the ability to capture extended drought recovery, thus underestimating the total carbon cost of drought. On the other hand, if models overestimate drought-year growth reduction, it is possible that they are misallocating NPP through time. From a carbon perspective, for example, a strong drought response may compensate for weak legacy effects, resulting in a realistic net carbon impact. Thus, a model might arrive at the “correct” productivity, but for the wrong reason. While that model might be able to reproduce contemporary productivity, these compensatory errors would likely produce poor predictions of future productivity, particularly under unprecedented environmental conditions. In addition, and in contrast to observations, the model ensemble produces similar patterns of growth recovery from drought across three distinct forest types and a climate gradient. These deficiencies suggest that models lack crucial representations and/or parameterizations of the physiological mechanisms driving diverse drought recovery features across different types of vegetation and climates. For instance, the weak drought legacy effects in models could be driven by lack of representation of non-structural carbohydrates; overly simplistic representation of forest dynamics and mortality, including reestablishment; and/or problems with excessively rapid LAI recovery, which may be unrestrained by functional sapwood requirements^43^. Nevertheless, while models underestimate the magnitude of drought legacy effects relative to observations, they simulate legacy effects in the right direction (i.e. NPP decreases). It is unclear what mechanisms drive this feature. It likely results from the downregulation of photosynthesis as water becomes less available, but both water availability (e.g. how soil moisture is simulated) and downregulation of photosynthesis are model-specific, The significant variation in drought legacy effect characteristics across models implies that model structure, or combination of model features, plays an important role in how different models simulate drought recovery. However, the complexity of current models makes it difficult to trace specific model behavior back to specific model choices/structure^44^. New approaches that transform the mathematical framework into matrix form may provide new opportunities to identify specific combinations of model structural characteristics that simulate more realistic drought recovery.

It is vital to address these potential model deficiencies, because climate change will likely strengthen the role of drought and consequent drought recovery as drivers of changes in terrestrial carbon cycling and storage. In particular, the frequency of extreme climate events is projected to increase, which raises the possibility that ecosystem drought recovery time may exceed the drought recurrence interval in certain critical zones^19^. The Amazon, for example, experienced an extreme drought in 2005 and was unable to recover full forest function before the onset of an even more extreme drought in 2010^23^. Furthermore, there is evidence that both ecosystem recovery times and the fraction of land undergoing recovery increased over the 20^th^ century, and it is likely that this trend towards longer and more spatially extensive drought recovery will continue^26^; although, CO_2_ fertilization effects may in some cases improve resiliency to recurring drought by shortening ecosystem recovery time^45^. Models must be capable of simulating realistic drought recovery in order to capture the exacerbated impact of drought on a recovering system, to anticipate potential tipping points resulting from systems unable to recover fully from persistently recurring droughts, and, thus, to produce reliable forecasts of global land carbon sink strength and consequent future atmospheric CO_2_ concentrations.

## Methods

### Models

To assess the ability of TBMs to simulate ecosystem response to, and recovery from, drought, we use simulation output from twelve models participating in the Multi-scale Synthesis and Terrestrial Model Intercomparison Project (MsTMIP)^46^. MsTMIP models are run using a common protocol that specifies driver data spatial and temporal resolution, and spin up conditions^46,47^. Additionally, MsTMIP defines a set of sensitivity simulations designed to isolate the impact of different drivers (climate, atmospheric carbon dioxide concentration, land cover change history, and nitrogen deposition) on simulated variables (e.g. carbon and energy fluxes, carbon pools). Time-varying simulations extend from 1901 to 2010. To isolate modeled vegetation response to climate extremes, we use model output corresponding to the climate-varying sensitivity simulation (SG1), in which atmospheric carbon dioxide concentration, land use, and nitrogen deposition are all held constant. Vegetation response was quantified by examining changes in modeled NPP.

### Identification of drought events

To identify climate extremes, we employ the Climatic Water Deficit (CWD)^48^ as a drought metric. CWD provides a measure of drought that accounts for water deficit at the plant level, rendering it a better indicator of plant water stress than more meteorologically-focused drought metrics, such as the Standardized Precipitation Index^49^ or Palmer Drought Severity Index^50^. CWD is commonly calculated in two ways: soil moisture minus potential evapotranspiration (PET) or precipitation minus PET. We define CWD as precipitation minus PET, because the use of soil moisture becomes problematic when working with model output due to the lack of standardization in how soil moisture is reported by models (e.g. the depth and number of soil layers).

To maintain consistency with the modeled environment, we use the same precipitation dataset that MsTMIP prescribes as driver data: CRU-NCEP^47^. PET is defined using a monthly, 1.0° gridded PET dataset covering 1948–2008 derived by Sheffield *et al*.^51^. This PET dataset was calculated using the Penman-Monteith algorithm and a meteorological forcing dataset that combines the NCEP-NCAR reanalysis product with several satellite-derived meteorological datasets, including Climatic Research Unit (CRU) climate variables, Tropical Rainfall Measuring Mission precipitation, and NASA Langley surface radiation budget. Thus, the Sheffield PET product is largely consistent with the forcing data prescribed to the models. Prior to analysis, the PET product was regridded to 0.5° using the nearest neighbor method in order to match the resolution of the model output and precipitation data. Monthly and annual CWD were calculated, and annual CWD was Z-scored to facilitate identification of extreme drought events. Extreme drought events were defined using the threshold CWD ≤ −2, or two standard deviations below the mean, which is a criterion commonly used to identify extreme droughts^21,49,52^.

### Lag time

To identify if there is a characteristic lag in plant productivity response to climate variability, we examine the correspondence between monthly CWD and NPP across the entire time series. For each grid cell, 61 correlations between the complete time series (1948–2008) of monthly de-seasonalized (grid cell monthly means removed) CWD and monthly de-seasonalized NPP are performed, with NPP offset from CWD by 0–60 months (e.g. a one month offset correlates NPP starting in month 2 with CWD starting in month 1). The optimum lag time is identified by finding the offset corresponding to the maximum correlation out of the set of 61 correlations. Here, the maximum correlation expresses the magnitude of NPP sensitivity to moisture anomalies, while the lag time describes the timescale of the NPP response. For instance, if NPP were driven primarily by concurrent CWD and, consequently, responded immediately to and in phase with CWD anomalies, we would expect to find no lag and a high sensitivity. Non-zero lags and/or lower correlations would suggest the NPP anomalies are influenced by other factors such as radiation (not captured by CWD), past climate, or drought recovery (which represents the decoupling of growth and climate).

We perform lag correlation analysis for each model individually and average results across models to obtain the model mean. Only grid cells containing significant correlations after adjusting for the false discovery rate^53^ are included in the model mean calculations. For clarity, and to examine potential seasonal and annual signals, lag times are binned by the following intervals: 0 months, 1 month, 2 months, 3–6 months, 7–12 months, 13–24 months, and >24 months.

### Quantification of drought response and recovery

We quantify the carbon cost of vegetation’s drought response and recovery as the difference between annual NPP and the climate-normal mean NPP. For each grid cell, the climate-normal mean NPP is calculated using only years of normal hydrologic conditions, i.e., −1 ≤ CWD ≤ 1. These NPP anomalies are calculated for the last year of a given extreme drought event (CWD ≤ −2) and the four subsequent years (producing a 5-year set of NPP anomalies). Extreme drought events followed by another extreme drought within four years are excluded from analysis in order to ensure that drought response did not obscure drought legacy effects. We evaluate all extreme droughts between 1948 and 2008, a period that captures historical extreme droughts such as the 1950s drought events across the US, the 2000–2004 drought in western North America, and the 2003 European heat wave. NPP anomalies corresponding to multiple drought events within a grid cell were averaged together but not across grid cells, preserving spatial heterogeneity. This analysis is performed for each model, and then averaged across all models to obtain the ensemble mean.

To assess the accuracy of modeled vegetation drought recovery, we performed a similar analysis using a different definition of NPP anomalies that facilitated comparison against an observational benchmark (refer to *Observational constraint* section below). Because NPP increases with water availability and decreases during drought, annual NPP and CWD are expected to be strongly positively correlated. In other words, higher values of CWD (wetter) correspond to higher NPP, on average, while lower values of CWD (drier) correspond to lower NPP. This relationship is strongest in water-limited regions, but it is significant across many temperature-limited grid cells as well (see Fig. S9 for model mean correlations). Accordingly, for grid cells exhibiting significant correlations between NPP and CWD (significant and *r* > 0.3; see Fig. S9 for the number of models that meet this criterion per grid cell), we define NPP anomalies as the percent difference between model simulated NPP and NPP predicted by CWD through a linear regression for each grid cell (Fig. S10), where a non-zero value indicates the degree to which NPP is operating outside of its typical relationship with climate.

We evaluate each model’s drought response and recovery by averaging all 5-year sets of NPP anomalies (drought year plus four subsequent years) corresponding to drought events. We chose the above *r* threshold to match the criterion used to select the tree ring chronologies comprising the observational benchmark^21^. Using different *r* thresholds for grid cell selection yielded similar results, although higher thresholds decreased the sample size considerably (Fig. S11). To calculate the ensemble model mean, the mean NPP anomalies from each model are averaged together, and the 95% confidence intervals are calculated by bootstrapping the model means (*n* = 5000). Thus, the confidence intervals around the ensemble model mean are a measure of model mean spread rather than model uncertainty. This analysis is first applied across all forested grid cells within the spatial domain, then across each of the following forest types separately: needle evergreen forest, broadleaf deciduous forest, and mixed forest.

### Observational constraint

Finally, to evaluate whether the models simulated drought recovery realistically, we use a previous study that identified drought legacy effects across tree ring chronologies^21^ as an observational benchmark. This benchmark comprises data from over 1000 sites across northern hemisphere forests and spans from 1948 to 2008. Although other available data products such as MODIS NPP and GIMMS NDVI have greater spatial coverage, they lack adequate temporal range (9 and 27 years overlapping with the CWD record, respectively). To facilitate comparison with this benchmark, analysis of drought events is confined to a spatial domain encompassing most of the sites analyzed by Anderegg *et al*.^21^: the contiguous United States (25°N–50°N, 125°W–60°W) and Europe (36°N–70°N, 10°W–36°E). Additionally, since the benchmark is based on tree ring chronologies, we analyzed only forested grid cells. Although modeled aboveground biomass is a closer proxy for tree ring growth, only five MsTMIP models report this variable, whereas NPP is reported by all twelve models. Across the five models that report both metrics, NPP and aboveground biomass are strongly correlated (*r* = 0.56) within the spatial domain of analysis.

### Code

All data processing and analysis was performed using MATLAB 2017B. The complete set of code used in the primary and supplementary analysis is available at https://www2.nau.edu/huntzingerlab/index.php/research/code/.

## Supplementary information

Supplemental Information

## Data Availability

Finalized MsTMIP data products are archived at the ORNL DAAC (http://daac.ornl.gov).
