# Supplementary material for: Land carbon models underestimate the severity and duration of drought’s impact on plant productivity
Source: Sci Rep. 2019 Feb 26;9:2758. doi: 10.1038/s41598-019-39373-1 (PMC6391443; doi:10.1038/s41598-019-39373-1)
Supplement: Supplementary file 1 — Supplemental Information [file 41598_2019_39373_MOESM1_ESM.pdf]

Supplemental for:

Land carbon models underestimate the severity and duration of drought's impact on plant productivity

Hannah R. Kolus[1*], Deborah N. Huntzinger[1], Christopher R. Schwalm[2], Joshua B. Fisher[3], Nicholas McKay[1], Yuanyuan Fang[4], Anna M. Michalak[4], Kevin Schaefer[5], Yaxing Wei[6], Benjamin Poulter[7], Jiafu Mao[8], Nicholas C. Parazoo[3], Xiaoying Shi[8]

[1]School of Earth and Sustainability, Northern Arizona University, P.O. Box 4099, Flagstaff, AZ 86011-5694, United States of America

[2]Woods Hole Research Center, Falmouth, MA 02540, USA

[3]Jet Propulsion Laboratory, California Institute of Technology, 4800 Oak Grove Dr., Pasadena, CA, 91109, USA.

[4]Department of Global Ecology, Carnegie Institution for Science, Stanford, CA 94305, USA

[5]National Snow and Ice Data Center, Cooperative Institute for Research in Environmental Sciences, University of Colorado, Boulder, Colorado, USA

[6]Environmental Sciences Division, Oak Ridge National Laboratory, Oak Ridge, TN 37831, USA

[7]NASA GSFC, Biospheric Sciences Lab., Greenbelt, MD 20771

[8]Environmental Sciences Division and Climate Change Science Institute, Oak Ridge National Laboratory, Oak Ridge, Tennessee 37831-6301, USA

* Corresponding author: (hrk37@nau.edu)

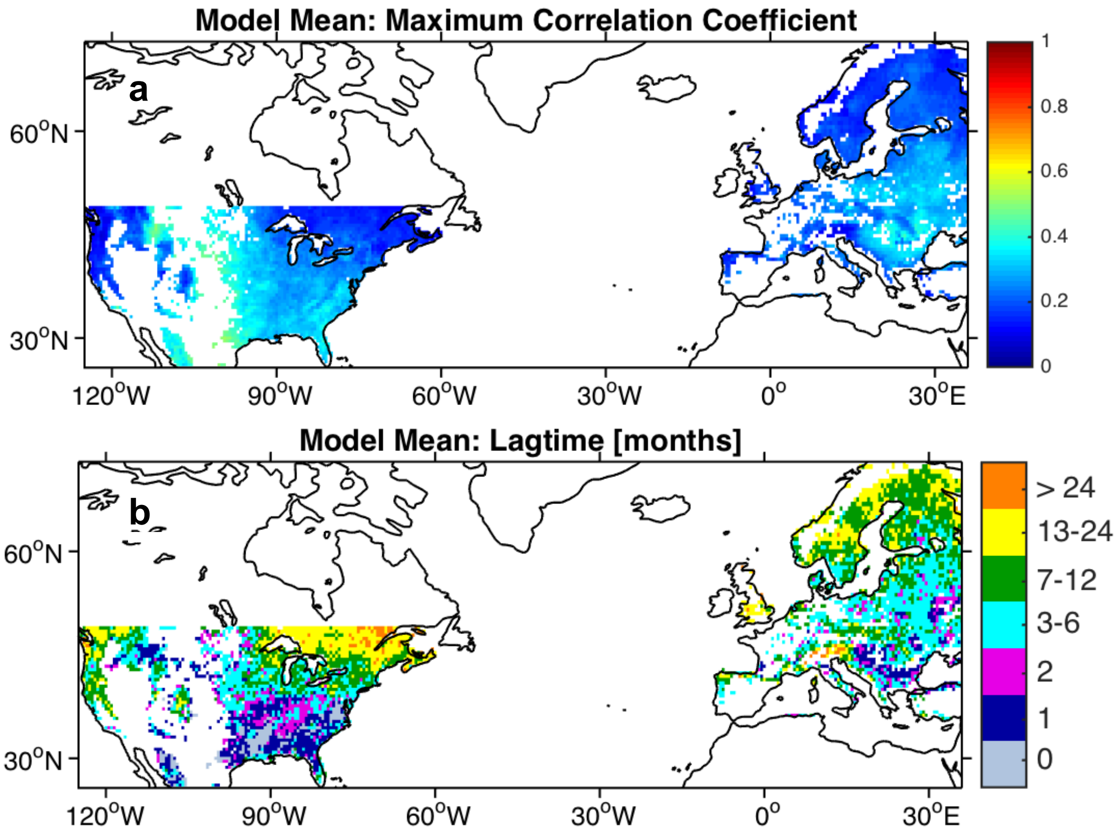

Figure S1 NPP sensitivity to precipitation anomalies. (a) Mean across models of maximum correlations between monthly de-seasonalized precipitation and monthly de-seasonalized NPP (maximum of 61 correlations calculated per grid cell, with an offset of 0 – 60 months between NPP and precipitation). (b) Lag time (offset) corresponding to the maximum correlation. Values have been binned. Averages for each grid cell were calculated using only significant correlations (FDR-adjusted $P < 0.05$) and their corresponding lag times per model.

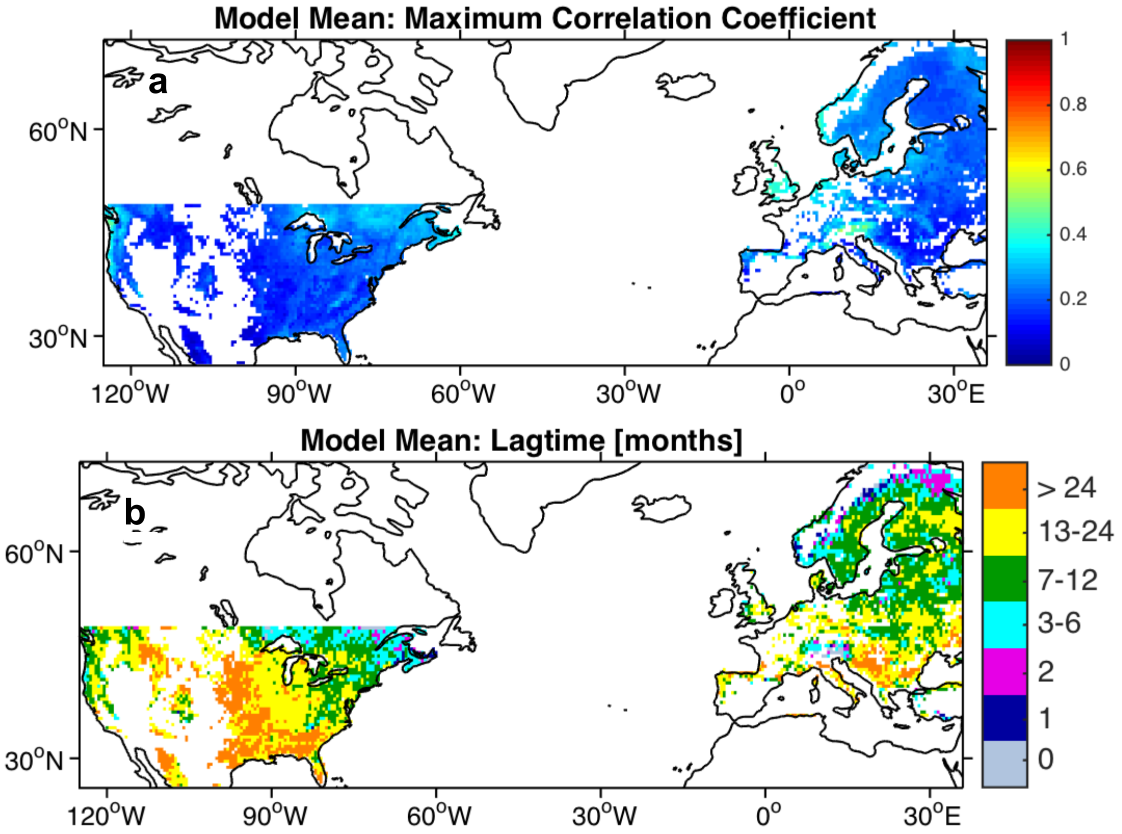

Figure S2 NPP sensitivity to temperature anomalies. (a) Mean across models of maximum correlations between monthly de-seasonalized temperature and monthly de-seasonalized NPP (maximum of 61 correlations calculated per grid cell, with an offset of 0 – 60 months between NPP and temperature). (b) Lag time (offset) corresponding to the maximum correlation. Values have been binned. Averages for each grid cell were calculated using only significant correlations (FDR-adjusted $P < 0.05$) and their corresponding lag times per model.

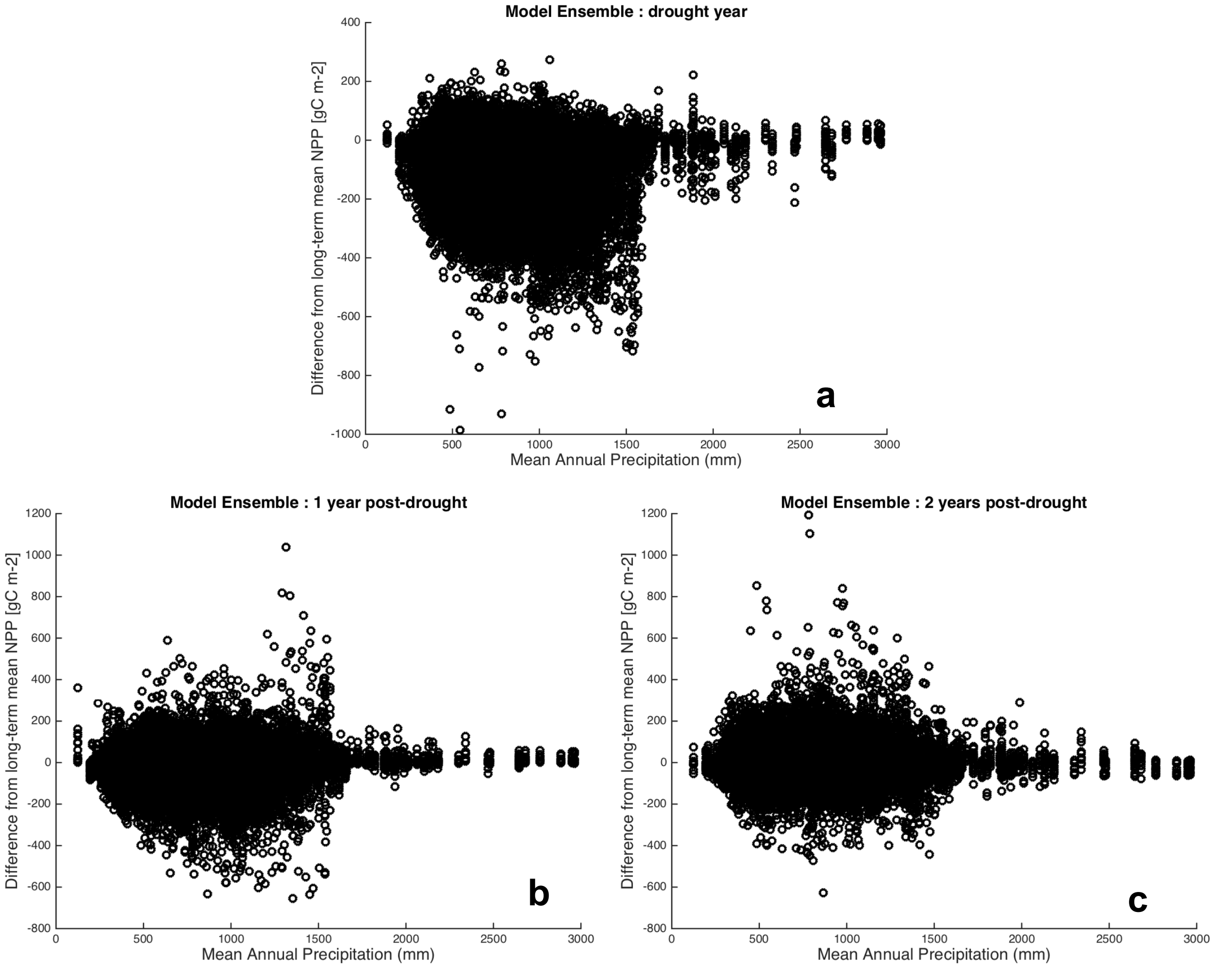

Figure S3 Relationship between mean climate and drought impact. The difference between NPP and the climate-normal mean NPP in (a) the drought year, (b) the first post-drought year, and (c) the second post-drought year is plotted against mean annual precipitation (an indicator of mean climate). Each circle represents the drought response of a grid cell corresponding to an individual model (i.e. no averaging across or within models).

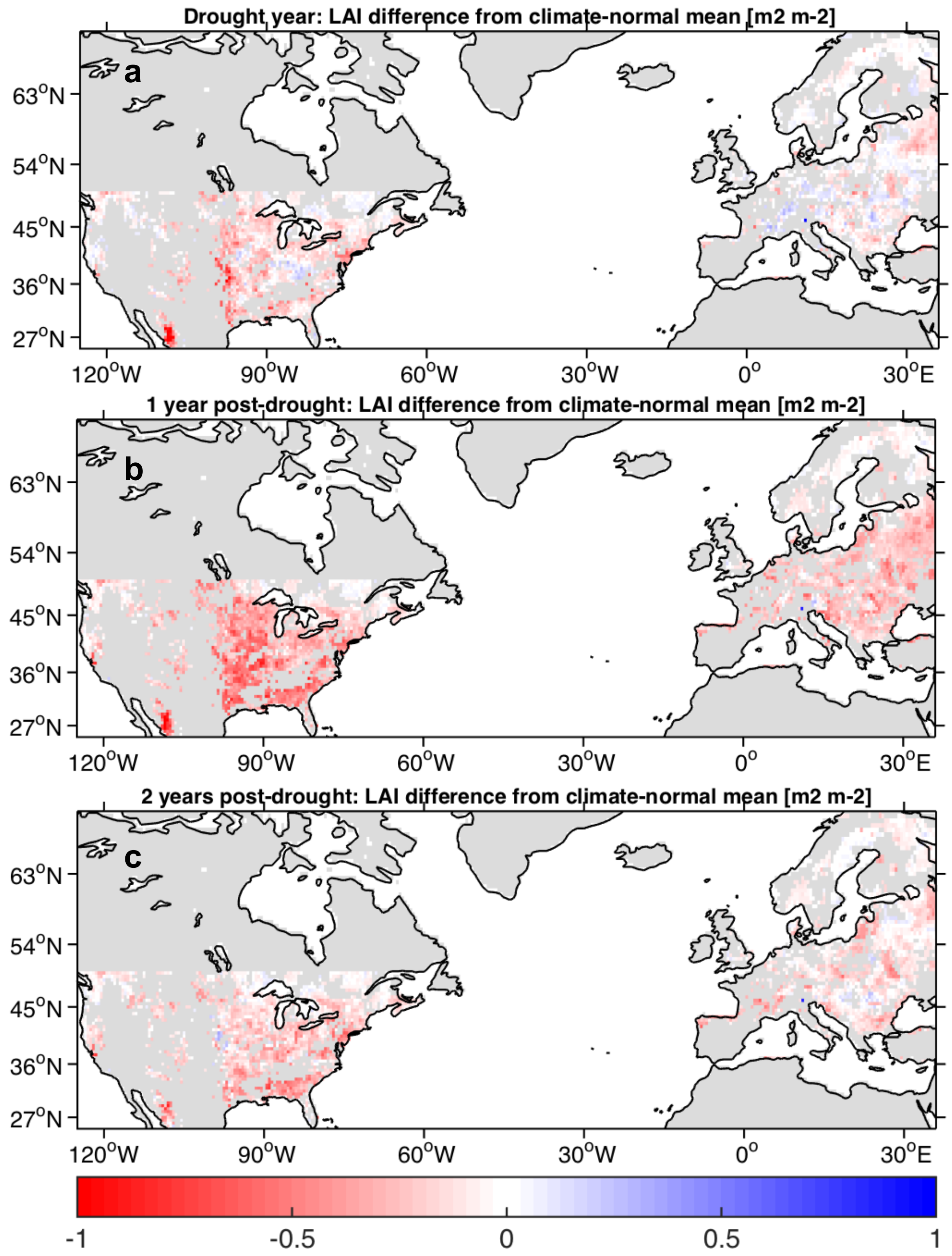

Figure S4 Spatial maps of the model mean LAI anomalies. Anomalies are defined as the difference from the climate-normal LAI of each grid cell (the average maximum LAI across years in which $-1 \leq CWD \leq 1$). LAI anomalies correspond to (a) the drought year, (b) one year post-drought, and (c) two years post-drought. Grey areas indicate pixels that experienced no drought events or that were excluded from analysis (outside spatial bounds, unforested grid cells).

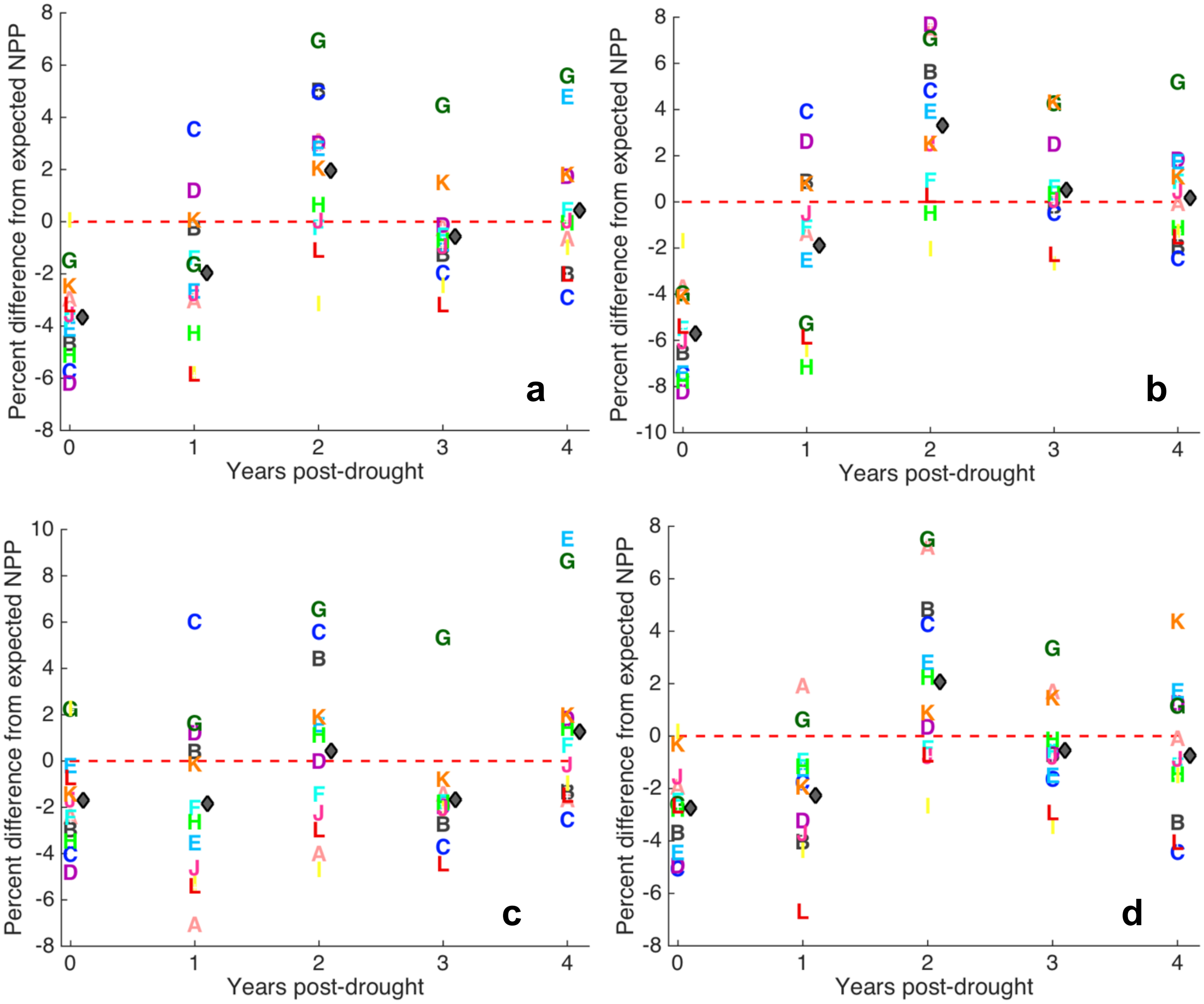

Figure S5 Individual model mean percent differences between NPP and expected NPP based on CWD. Differences are averaged over (a) all forested grid cells with the region of analysis, (b) evergreen needle forest, (c) broadleaf deciduous forest, and (d) mixed forest. Colored letters indicate results from individual models (each model has a unique color and letter, which are consistent across all plots). Diamonds indicate the mean across models. Note the different y-axis scales.

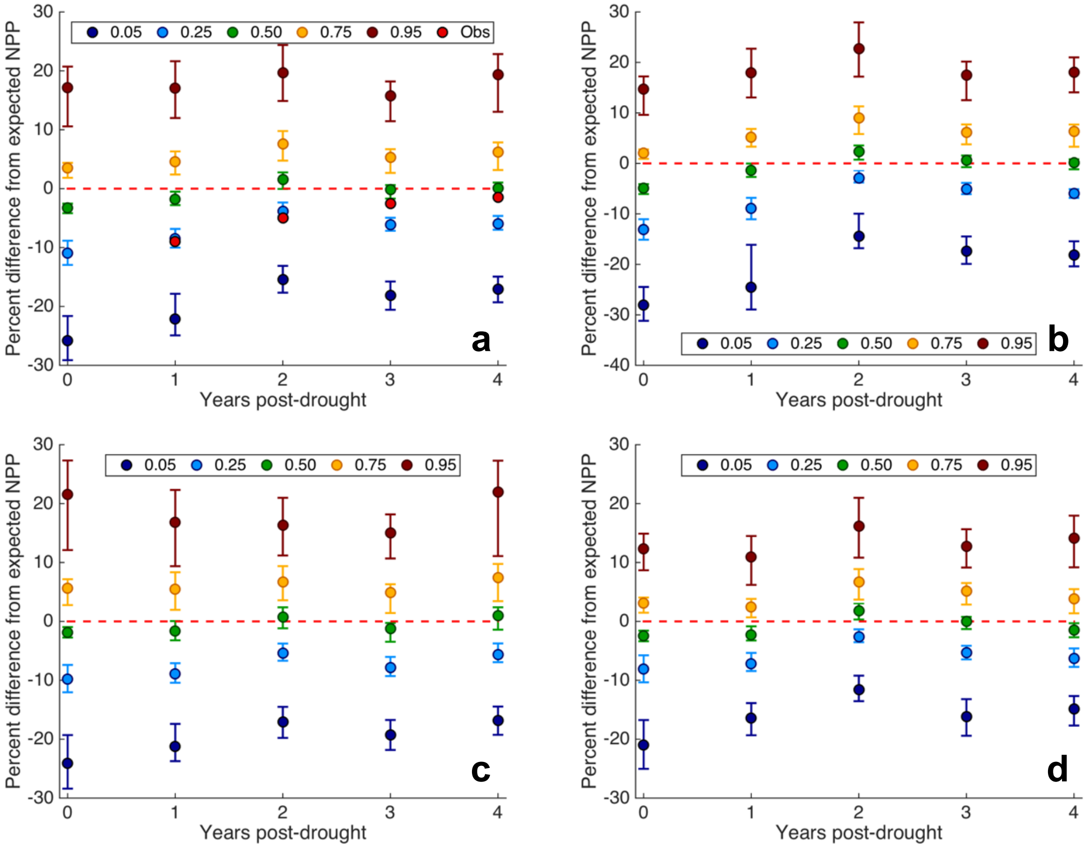

Figure S6 Percentiles of percent differences between NPP and expected NPP based on CWD. The 5th, 25th, 50th, 75th, and 95th percentile legacy effects were identified for each model over (a) all forested grid cells with the region of analysis, (b) evergreen needle forest, (c) broadleaf deciduous forest, and (d) mixed forest. These values were then averaged across models, and error bars indicate the 95% confidence intervals calculated by bootstrapping (n = 5000) the model percentiles. In (A), the red circles indicate an observational benchmark derived from tree rings (Anderegg et al., 2015). Note the different y-axis scales.

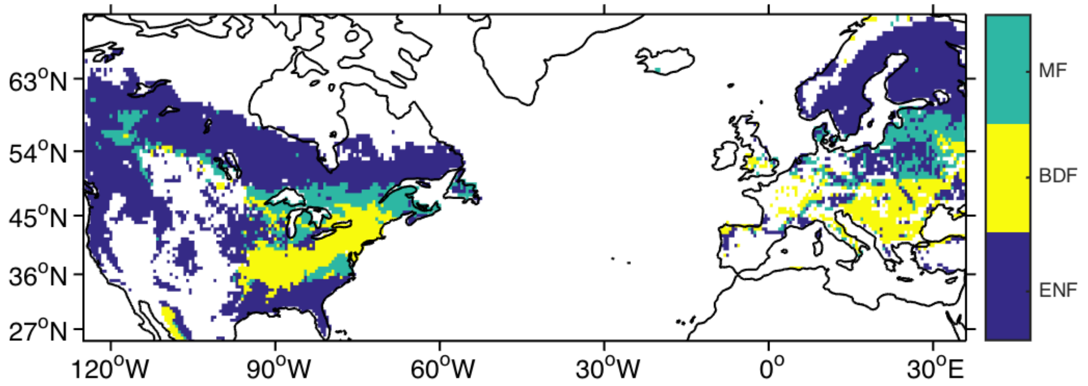

Figure S7 Map of forest types. Mixed forest (MF), broadleaf deciduous forest (BDF), and evergreen needle forest (ENF). White areas indicate non-forested regions.

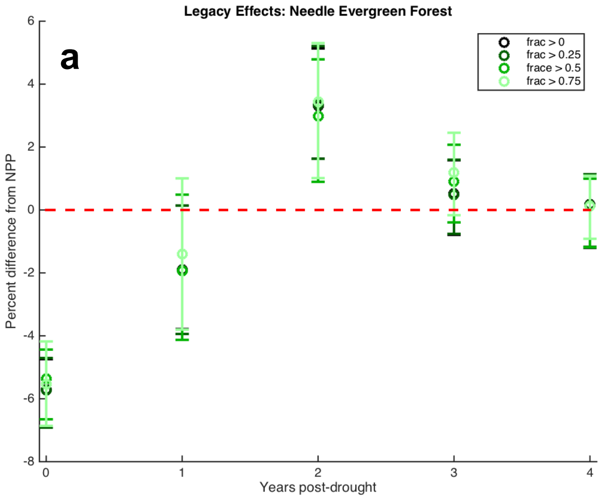

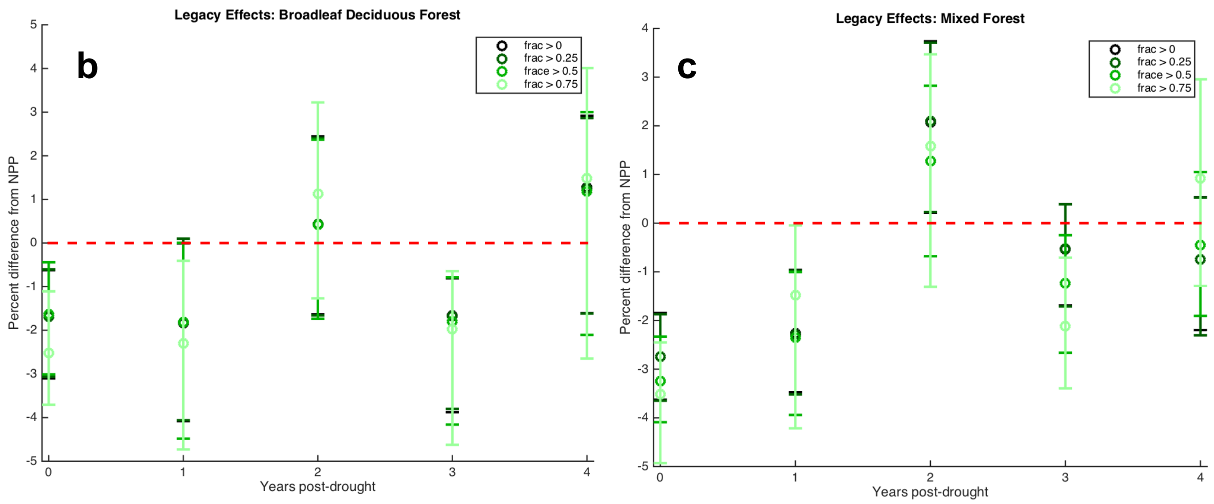

Figure S8 Effect of pixel purity on drought response and legacy effects. Percent differences between NPP and expected NPP based on CWD are averaged over (a) evergreen needle forest, (b) broadleaf deciduous forest, and (c) mixed forest using different thresholds of dominant forest type fraction (> 0, > 0.25, > 0.5, and > 0.75). For example, grid cells that meet the highest threshold must contain at least 75% cover characterized by the given forest type. All circles indicate the mean across models, and error bars indicate the 95% confidence intervals calculated by bootstrapping (n = 5000) the model means. Note the different y-axis scales.

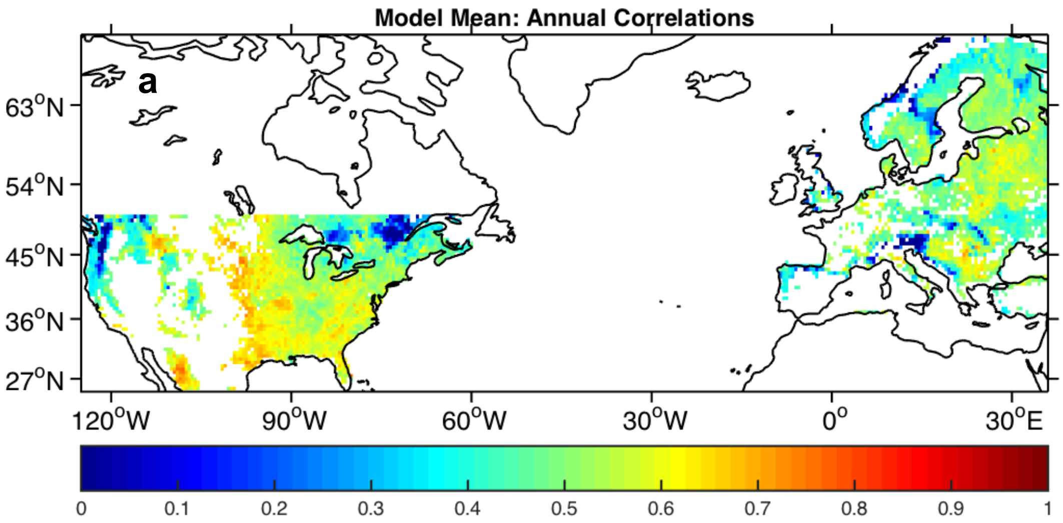

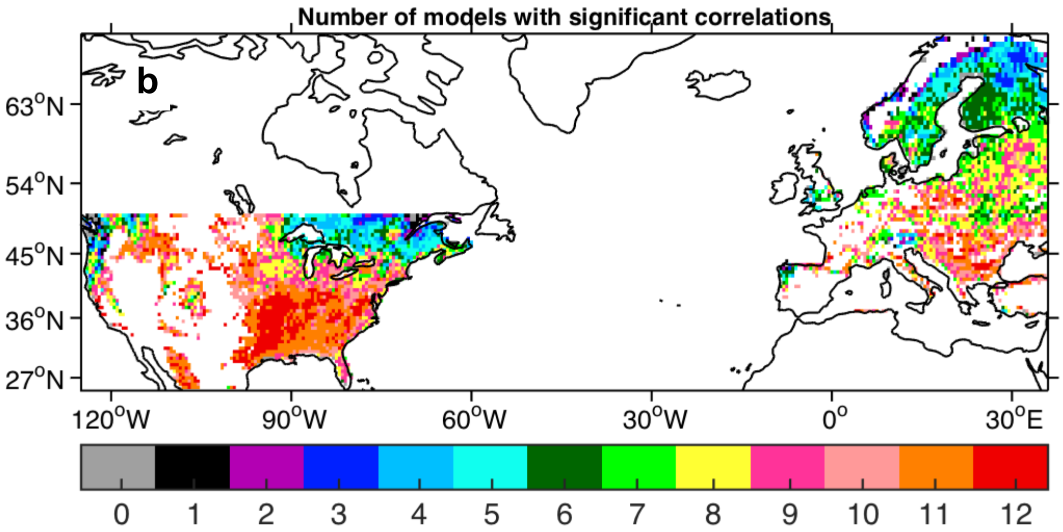

Figure S9 Model mean correlations between annual NPP and CWD. (Aa) Mean across models of the correlations between annual CWD and NPP. The model mean was calculated using only grid cells exhibiting significant correlations from each model. (b) The number of models exhibiting significant correlations per grid cell.

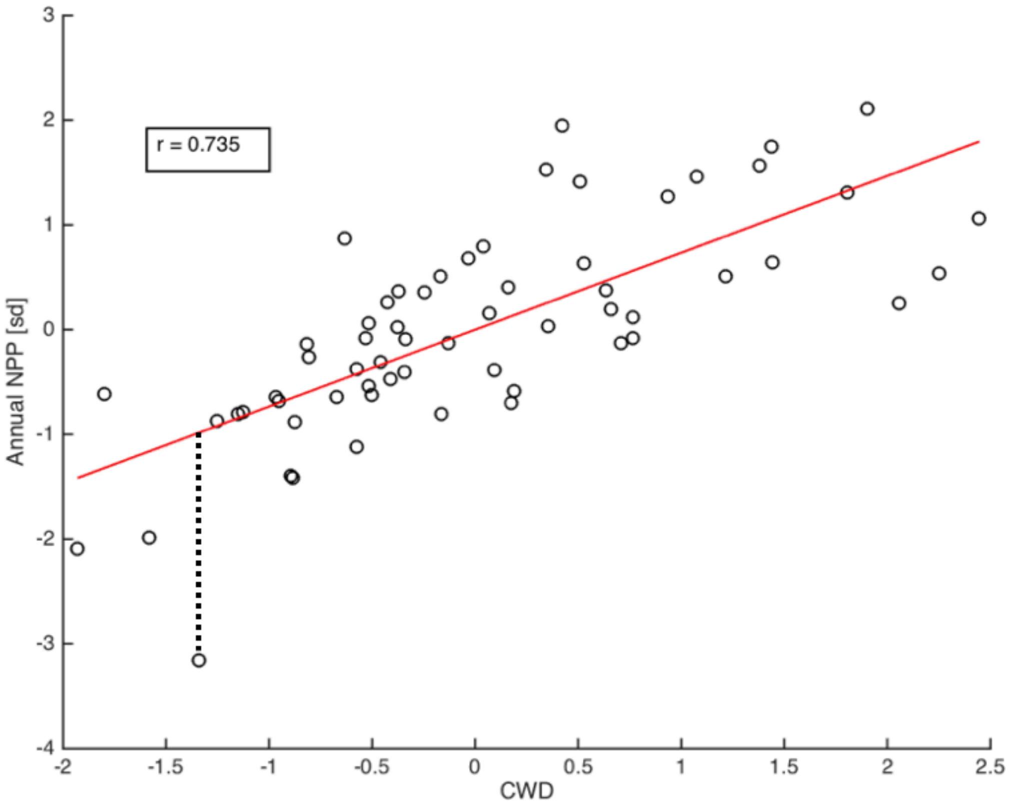

Figure S10 Example linear regression between annual CWD and NPP. The red line is the best-fit line. The box indicates the correlation between NPP and CWD. The dashed line indicates the difference between the NPP in a given year minus the NPP predicted by CWD. Data comes from a grid cell in CLM.

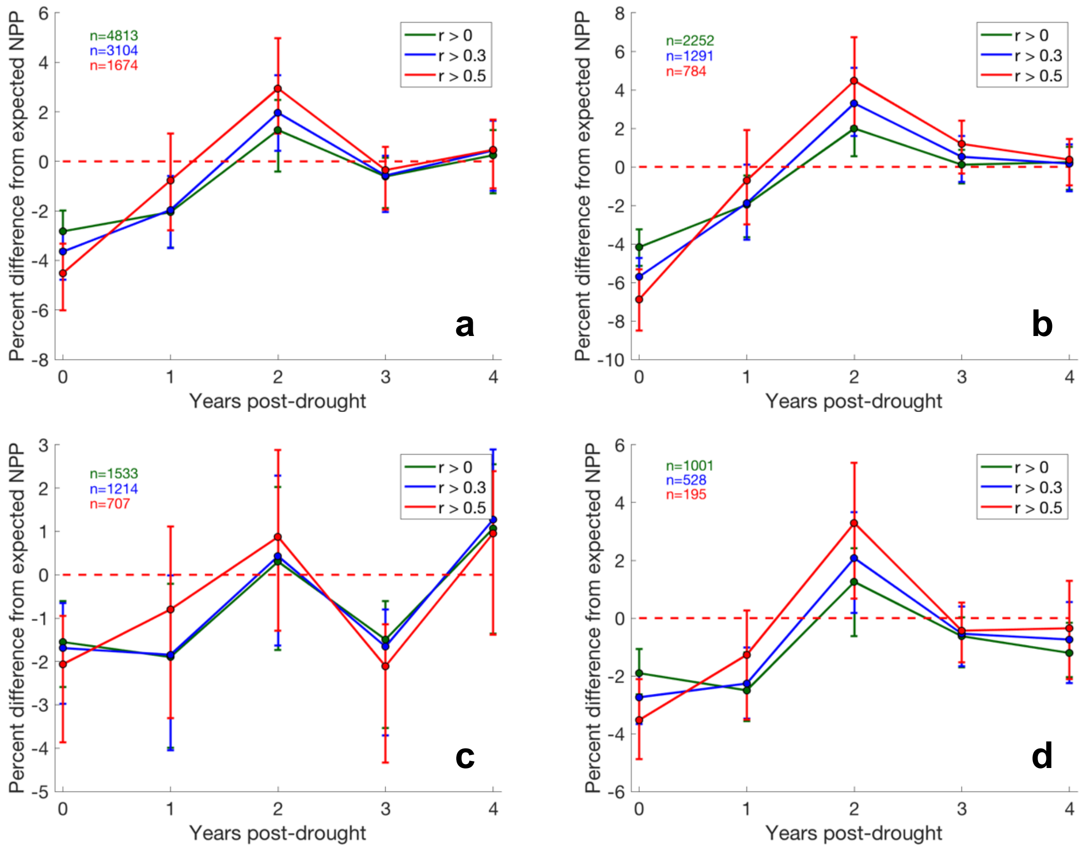

Figure S11 Percent difference between NPP and expected NPP based on CWD across r thresholds. Differences are averaged over (a) all forested grid cells with the region of analysis, (b) evergreen needle forest, (c) broadleaf deciduous forest, and (d) mixed forest. Thresholds of r > 0, r > 0.3, and r > 0.5 are shown (r > 0.7 yielded sample sizes of 0 for some models). The sample size n indicates the median number of grid cells that meet the threshold and forest type criteria across the models. Note the different y-axis scales.
